# Kidney transplantation in abnormal bladder

**DOI:** 10.4103/0970-1591.33728

**Published:** 2007

**Authors:** Shashi K. Mishra, V. Muthu, Mohan M. Rajapurkar, Mahesh R. Desai

**Affiliations:** Department of Urology and Nephrology, Muljibhai Patel Society for Research in Nephrourology, Muljibhai Patel Urological Hospital, Nadiad - 387 001, Gujarat, India

**Keywords:** Abnormal bladder, augmentation, transplantation

## Abstract

Structural urologic abnormalities resulting in dysfunctional lower urinary tract leading to end stage renal disease may constitute 15% patients in the adult population and up to 20-30% in the pediatric population. A patient with an abnormal bladder, who is approaching end stage renal disease, needs careful evaluation of the lower urinary tract to plan the most satisfactory technical approach to the transplant procedure. Past experience of different authors can give an insight into the management and outcome of these patients. This review revisits the current literature available on transplantation in abnormal bladder and summarizes the clinical approach towards handling this group of difficult transplant patients. We add on our experience as we discuss the various issues. The outcome of renal transplant in abnormal bladder is not adversely affected when done in a reconstructed bladder. Correct preoperative evaluation, certain technical modification during transplant and postoperative care is mandatory to avoid complications. Knowledge of the abnormal bladder should allow successful transplantation with good outcome.

Structural urologic abnormalities resulting in dysfunctional lower urinary tract leading to end stage renal disease may constitute 15% patients in the adult population and up to 20-30% in the pediatric population.[1] An abnormal urinary bladder is no longer a contraindication to renal transplantation. With appropriate selection, patient and graft survival is not significantly different from that of the general transplant population.[2] We summarize the current literature available to evaluate abnormal bladder during pretransplant workup, discuss the intraoperative and postoperative management of problems during transplantation.

Normal and abnormal bladder - Normal urinary bladder stores urine at low pressure, does not leak and completely empties by natural voiding. Errando *et al*.[2] defined a defunctionalized bladder as one where the urinary output is less than 300 ml in 24h. Others have defined it as a bladder that is decompressed and has not been used for several years.[3] If voiding is normal before the patient develops oligoanuria, the bladder will develop normal capacity within a few weeks after transplantation.[4] When intravesical pressures exceed 40 cm H_2_O, ureteral transport of urine ceases[5] and it is mandatory to maintain intravesical pressure less than 30 cm H_2_O during filling to prevent upper urinary tract damage. A urinary bladder may be abnormal because of neuropathy, bladder outlet obstruction (posterior urethral valve, urethral stricture), acquired voiding dysfunction (Hinman syndrome), acquired bladder disease (interstitial cystitis, post-radiotherapy changes, fibrosis from intravesical chemotherapy), perivesicular scar (pelvic hematoma, prior ipsilateral transplant) and augmented bladders.

## MANAGEMENT

Management of these bladders during transplantation should include specific strategies during pre, peri and post-transplant period. It is reasonable that a patient with an abnormal bladder, who is approaching end stage renal disease (ESRD), needs careful evaluation of the lower urinary tract to plan the most satisfactory technical approach to the transplant procedure. Evaluation should be made well in advance,[6] to anticipate and work out solutions to problems that may be encountered later on. In our series of 1570 renal transplants, we encountered 44 patients who had dysfunctional lower urinary tract. Causes for abnormal bladder included 33 posterior urethral valve (PUV), six neurogenic bladder, one non-neurogenic bladder. One patient had history of ureteric reimplantation done twice in the past. Ileal conduit was present in two patients and one patient had solitary left kidney with ureterostomy. Overall patient and graft survival rate in these patients was 72.7% and 59.1% at two years follow-up.

### Pretransplant evaluation

Evaluation – Preoperative evaluation includes history of native kidney disease, detailed physical examination with special attention to abdominal scars, stomas and catheter. Investigations include urine cultures, abdominal sonography, micturating cystourethrogram (MCUG), uroflow and urodynamic studies. For patients who have sufficient urine output, a voiding diary that documents urinary continence, voided volumes and times of voiding is helpful to determine the need for urodynamic studies.

Urodynamic studies in transplant patients[7] indicate that, higher leak point pressures are associated with a lesser chance of success in patients with PUV or obstructive uropathy. A bladder capacity of less than 100ml or voiding pressures of more than 100 cm H_2_O may predispose to complications after transplantation and are best served by bladder augmentation.[8] Defunctionalized bladders having low pressures during the filling phase do well after renal transplantation. Poor complaint bladder requires rehabilitation before transplantation to avoid allograft injury. Anticholinergic drugs can be used to decrease bladder filling pressure and unstable contractions.

Micturating cystourethrogram is recommended in children who have more congenital urological abnormalities.[9] It should be done when there is a history of lower urinary tract symptoms. It can diagnose the presence of vesicoureteric reflux (VUR), post-void residual urine, PUV and urethral strictures, which can have a bearing on the treatment plan to the bladder.

## PRETRANSPLANT ISSUES

### CISC

Patients with abnormal bladder have to be sterile at the time of transplant. All culture-positive urinary tract infections should be treated with appropriate antibiotics; patients keeping large residual urine are educated to do double and frequent voiding. Patients requiring CISC should be taught correct technique and motivated to do so if not anuric, to increase compliance. A native urethra may be unsuitable for CISC in children with anatomical anomalies that lead to difficult or painful catheterization. When faced with this scenario, it is usually critical to ensure compliance with intermittent catheterization by employing alternate Mitrofanoff neourethra. A continent abdominal stoma using the Mitrofanoff principle gives reliable results in children and is well tolerated. This facilitates catheterization easy, convenient and pain-free.[10] The appendix is eminently suitable for this purpose but the ureter provides a satisfactory alternative in selected cases. When neither is available, alternative techniques for constructing a catheterizable continent channel may be considered.[11]

### VUR

Refluxing system in the immunosuppressed transplant patient may contribute to urinary tract infection (UTI) especially when associated with voiding dysfunction. Vesicoureteric reflux per se without voiding dysfunction post transplant does not correlate with subsequent graft function, hypertension, rejection or the number of UTI episodes.[12] When VUR exists, preoperative evaluation should be performed to rule out bladder outlet obstruction or spastic bladder. Neurogenic bladders causing severe reflux may require augmentation to produce low-pressure reservoir. If the native ureters have not been reimplanted, both native dilated ureters can serve as vascularized patch grafts for augmentation cystoplasty. The outcome of renal transplantation in patients with high-grade reflux may be disappointing due to postoperative UTI. Reimplantation prior to transplant represents over-treatment and may cause perivesical changes that may complicate the pending transplant. Subureteral injection has been used frequently with encouraging results in VUR.[13] However, its use in high-grade reflux is controversial because of its reduced success rates. Postoperative obstruction is a rare possibility which can be ignored in anuric patients. For high-grade reflux, we prefer to preserve the native kidneys, which facilitate dialysis and may provide ureteral tissue for bladder augmentation. Appropriate surgical intervention should be done for unremitting potential source of infection especially in presence of documented symptomatic UTI either in pre or post-transplant period.[14]

### Pretransplant nephrectomy

Native kidney nephrectomy is seldom required prior to transplantation. Nevertheless the indications are: pyelonephritis, renin-mediated hypertension, malignant disease and unresolving severe nephrotic syndrome. Large polycystic kidneys can be removed prior to transplantation. We did 14 pretransplant nephrectomies for recurrent UTI in the 44 cases of abnormal bladder operated upon.

## PERITRANSPLANT MANAGEMENT

Peritransplant management should consist of bladder dynamics correction if possible or bypass of the lower urinary tract. The bladder, which contributed to the destruction of the native kidneys, may threaten a subsequently placed renal allograft.[15] In the past, urinary diversion was the usual approach to bypass the abnormal bladder. With the use of augmentation cystoplasty and CSIC in small-volume poor-complaint bladder, a low-pressure and complaint reservoir can be created that can protect the upper urinary tract and restore the functional lower urinary tract.[16]

### Transplant in ureterostomy

There are reports of successful transplantation in patients with long-standing cutaneous ureterostomies.[17–19] Available data suggests that a preexisting native cutaneous ureterostomy may serve as a conduit for graft ureteral drainage in select patients with excellent long-term function. We had an experience of solitary kidney patient where pretransplant cystectomy was done for recurrent pyocystitis. We transplanted graft in native kidney fossa after nephrectomy with graft ureter anastomosed to native ureterostomy. He had smooth postoperative recovery. Patient had multiple episodes of pyelonephritis and UTI during two years follow-up. The use of the donor ureter to create a cutaneous ureterostomy at the time of renal transplantation has also been reported.[20] Cutaneous ureterostomy is performed by suturing the edges of the distal end of the ureter to the skin. Complications frequently seen in these patients are pyelonephritis, urosepsis, stomal retraction, stomal stenosis and uretero-ureteric anastomotic stricture. There is also the need for repeated stomal dilatation to prevent functionally significant stenosis. However, both the forms of ureterostomy are feasible and should be considered as one of the options available to the surgeon in selected cases.

### Transplant in augmented bladder

In 1982 Marshall *et al*.[21] reported on the first augmentation cystoplasty after kidney transplantation in a man and, in 1984, Stephenson *et al*.[22] reported the first pediatric kidney transplant draining into an augmented bladder. Hatch *et al*.[23] reported the largest retrospective pediatric series including both patients with bladder augmentation (n=17) and urinary diversion (n=13). The graft survival was not significantly different for augmentation and diversion groups (78% vs. 46%), but it definitely favored the augmented group. Nahas *et al*.[24] reported five-year survival of 78% in 24 patients with abnormal bladder, 21 of whom had undergone enterocystoplasty. Urinary tract infection occurred in 56% and 32% required hospitalization. Similarly, there was no statistical difference between patients with augmentation or diversion as reported by Rischmann *et al*.[25] On the other hand Alfrey *et al*.[26] reported adverse outcomes in augmented bladder. In three of the eight patients, where kidney was transplanted along with augmentation, each had severe UTI. One died, one lost the graft and another was being considered for an incontinent diversion.

Fontaine *et al*.[27] suggested reconstructive surgery well before transplantation to avoid deleterious impact of immunosuppressive drugs on the healing process.

Most authors have performed the augmentation procedure before the transplantation. Though this approach is safe, it presents management dilemma in the rare few who are anuric. Cycling can be done in augmented bladder with reservoir filling twice a day with 300 ml of normal saline, to maintain adequate bladder volume and to remove enteric secretions that can accumulate and become a source of obstruction and infection.[28]

### Transplant in valve bladder

Children with PUV present management dilemma for augmentation. Bladder that appears inadequate before renal transplant may behave normally once the polyuria resolves. On the other hand, poor-compliance bladder for a given bladder volume may contribute or accelerate renal failure.[29] Salomon *et al*.[30] reported worse graft survival in children with PUV with symptomatic voiding dysfunction than those without. One other study concludes that limited intervention approach in PUV patients resulted in better outcome than extensive urologic procedures.[31] At present, however, no clear-cut recommendation can be made in patients of PUV in view of lack of controlled studies. The majority of the patients with PUV done at our institute had either normal bladder (66.6%) or large capacity bladder (30.3%) with no need for augmentation felt.

### Transplant in ureterocystoplasty

Sheldon *et al*.[15] proposed that the ureter should be used to augment bladder wherever possible, which has possible advantages of no mucus secretion, metabolic abnormality and use of urothelium for augmentation. Nahas *et al*.[32] analyzed eight patients who underwent renal transplantation following bladder augmentation with dilated ureter with mean follow-up of 50 months. All his patients remained continent. None of the grafts were lost and the most common complication was asymptomatic urinary tract infection. Ureterocystoplasty combines the benefits common to all enterocystoplasties without adding to complications or risks. The use of urothelial-lined biomaterial for augmentation avoids the potential complications of gastro- or enterocystoplasty, which are especially dangerous in transplant patients.

### Transplant in Intestinal conduit

Transplants into ileal conduit usually result in high surgical complication rate although with good graft survival rate.[23,33] Neild *et al*.[34] reported experience of 12 transplants on ileal conduit. The patients had good graft function initially but five-year graft survival was 56% and, for five of the seven that had failed, recurrent UTI was a significant factor. Conduit stoma should be created at least six to eight weeks before renal transplantation.[35] Both our patients with ileal conduit done in the early 1990s had a stormy postoperative course with pyelonephritis and graft loss within one year post transplant.

### Transplant in continent reservoir

There are case reports of transplantation into continent urinary diversion with good short-term results. Use of Kock, Mainz and Indiana pouch has been described before the transplantation.[36] Problems with obstruction and infection are common in these patients and require close observation.[37] Most patients with continent diversion empty by CISC. Although this results in virtually universal bactiuria, the safety of CIC and renal transplantation has withstood the test of time.[38,39]

## PEROPERATIVE MANAGEMENT

As a protocol, all our abnormal bladders are on suprapubic percutaneous catheter drainage during the peroperative and two weeks postoperative period. After the patient has recovered from the surgical trauma, he is put on bladder training or CISC as per the bladder dynamics. Barry *et al*.[40] found that defunctionalized bladder if filled to capacity, by gravity at the beginning of the case, can be further filled later, after vascular reconstruction. If Mitrofanoff or other continent stoma has been created, an extended Gibson's incision lateral to the stoma or in the contralateral lower abdomen will allow successful dissection. Allograft should be placed on the same side as the conduit to shorten the distance between the two, permitting linear urinary flow.[35]

MacKinnon *et al*.[41] described extravesical techniques that are applicable to the small bladder. Salvatierra *et al*.[42] described the use of Le Duc-Camey uretero-ileal implantation technique to the transvesical ureteroneocystostomy in patients with small, defunctionalized urinary bladders. Development of a submucosal tunnel for ureteroneocystostomy is usually easier in the bladder rather than the patch used for augmentation, with possible exception of the stomach.[41] A single-layer uretero-ileal anastomosis rather than two layers may prevent the development of uretero-ileal anastomotic strictures[43] in conduit diversion. We are routinely using Lich Gregoir ureteroneocystostomy for the thick-walled bladder with long submucosal tunnel without problems so far. For thick-walled bladders, it is safer to keep double J stent for two weeks to allow proper healing of the ureteroneocystostomy.

It is important to know the blood supply of the augmented patch so as not to interfere with it during the renal transplant procedure. For example, if the right gastroepiploic vessel is preserved for gastrocystoplasty, the surgeon would need to be aware that the vascular pedicle will be in the right retroperitoneum. If large or small bowel has been used for the cystoplasty, the vascular pedicle will enter the augmented bladder at or near its dome.[43]

## POSTOPERATIVE MANAGEMENT

Urinary tract Infection – Patients with conduit, augmentation and reservoir present a special problem because their urobowel is colonized with bacteria. If the patient is asymptomatic and pyelonephritis does not ensue, bactiuria does not require treatment. An exception is colonization with urea-splitting organisms such as Proteas mirabilis, because this may lead to formation of struvite stones. Urinary tract infection directly contributed to graft loss in patients with abnormal bladders but caused no consequences in those with normal bladders.[15] Symptomatic UTI (63%) are common in the first three months of transplantation.[40] Neild *et al*.[34] suggested use of prophylactic antibiotics for the first six months that halved the subsequent incidence of urinary tract infection. All our patients are on cotrimoxazole / trimethoprim prophylaxis for at least one year. If UTI recurs or increases in frequency, then a cause must be sought with proper evaluation. A plain radiograph is essential to look out for stones in native or transplant kidneys or in the bladder or urinary diversion. Seventy per cent of the abnormal bladders transplanted in our series had at least one documented UTI episode.

Intermittent self-catheterization - This is safe and effective for patients with a poor flow rate who fail to empty the bladder. This is possible in patients with normal urethra and cooperative patients. When not practical, Mitrofanoff stoma can be made to establish suprapubic drainage. When bowel segments are used, voiding using CSIC does not increase the risk of UTIs, even in the immune-compromised patients.[28] Clean self intermittent catheterization can be started during follow-up if there is residual volume after double micturition. Six patients of neurogenic bladder who were transplanted in our series were on CISC to evacuate bladder. One patient had recurrent UTI after CISC and he died of septicemia two years post transplant. On the contrary, one patient on CISC has normal renal parameters and is on CISC every four hourly for the last 16 years!

Clean self intermittent catheterization with Tieman catheter according to the penile urethral caliber is usually atraumatic if performed cautiously. Washing the hands and catheter with soap and application of lubricant (topical xylocaine jelly) is required prior to insertion. We recommend catheter to be reused for a period of three weeks before discarding. The catheter is washed with soap and water before and after the procedure and stored in a plastic container.

Other complications- Electrolyte abnormalities can occur depending on the bowel segment used. Acidosis should be treated because of its contribution to the metabolic bone disease. Mucus production inherent to the use of bowel can be dealt with by daily bladder irrigations and use of alkalizers. Megaloblastic anemia associated with use of distal ileum requires replacement with vitamin B12. Bladder or upper tract stones occur in 8-52% of patients with bladder augmentation.[34,44] Patients of continent diversion having large bowel have risk of adenocarcinoma developing at the anastomosis. Careful surveillance is indicated in this group, as chronic immunosuppression may increase the risk of cancer.[45]

## CONCLUSION

An abnormal bladder is no longer a contraindication for renal transplant. The outcome of renal transplant in abnormal bladder is not adversely affected when done in a reconstructed bladder. Proper preoperative evaluation, certain technical modifications during transplant and postoperative care is mandatory to avoid complications. Knowledge of the abnormal bladder should allow successful transplantation with good outcome. Graft implantation in the native bladder is always preferred. If the native bladder is unsuitable, then the graft may be drained into augmented bladder (preferably), a continent diversion or into an enteric conduit. Native dilated ureter should be preserved during pretransplant period for possible use of bladder correction by ureterocystoplasty, which has advantages of no mucus secretion and metabolic abnormality. Routine antibiotic prophylaxis and voiding using CSIC does not increase the risk of UTI, even in the immunocompromised patients.
